# Polarization Characterization of Porous Particles Based on DDA Simulation and Multi-Angle Polarization Measurements

**DOI:** 10.3390/ma17081718

**Published:** 2024-04-09

**Authors:** Shuan Yao, Heng Zhang, Nan Zeng, Hui Ma, Honghui He, Yuelu Jiang

**Affiliations:** 1 Guangdong Research Center of Polarization Imaging and Measurement Engineering Technology, Shenzhen Key Laboratory for Minimal Invasive Medical Technologies, Institute of Biopharmaceutical and Health Engineering, Tsinghua Shenzhen International Graduate School, Tsinghua University, Shenzhen 518055, China; ysa21@mails.tsinghua.edu.cn (S.Y.); zhangh.2020@tsinghua.org.cn (H.Z.); mahui@tsinghua.edu.cn (H.M.); he.honghui@sz.tsinghua.edu.cn (H.H.); 2 Department of Physics, Tsinghua University, Beijing 100084, China; 3 School of Environment, Tsinghua University, Beijing 100084, China; jiang.yuelu@sz.tsinghua.edu.cn

**Keywords:** porous particles, light scattering, polarization, discrete dipole approximation

## Abstract

Porous suspended particles are hazardous to human health due to their strong absorption capacity for toxic substances. A fast, accurate, in situ and high-throughput method to characterize the microporous structure of porous particles has extensive application value. The polarization changes during the light scattering of aerosol particles are highly sensitive to their microstructural properties, such as pore size and porosity. In this study, we propose an overlapping sphere model based on the discrete dipole approximation (DDA) to calculate the polarization scattering characteristics of porous particles. By combining scattering calculations with multi-dimensional polarization indexes measured by a multi-angle polarized scattering vector detection system, we achieve the identification and classification of pore-type components in suspended particles. The maximum deviation based on multiple indexes is less than 0.16% for the proportion analysis of mixed particles. Simultaneously, we develop a quantitative inversion algorithm on pore size and porosity. The inversion results of the three porous polymer particles support the validity and feasibility of our method, where the inversion error of partial particles is less than 4% for pore size and less than 6% for porosity. The study demonstrates the potential of polarization measurements and index systems applied in characterizing the micropore structure of suspended particles.

## 1. Introduction

Aerosols are defined as suspensions of liquid or solid particles in air. As an important component of the Earth’s atmosphere, they strongly impact climate change and environmental pollution [1]. On the one hand, aerosol particles play a significant role in Earth’s radiative balance by absorbing and scattering the incoming solar radiation and surface longwave radiation and changing the lifetime and optical characteristics of clouds [2,3]. On the other hand, in recent years, aerosol particles have become one of the leading causes of urban air pollution. Some inhalable particles may cause respiratory disease by depositing in the human bronchus and lung, and even damage the heart and brain through the bloodstream [4,5]. Certain particles deviate from regular spherical morphology and have intricate porous structures among numerous categories of suspended aerosol particles. These suspended particles are more likely to induce severe health hazards to humans due to their specific surface and strong absorption capacity for toxic and harmful substances in the atmosphere. This study is committed to detecting, identifying, and characterizing aerosol particles based on the polarization optical method, especially focusing on porous particles with complex morphology and structure.

Various methods have been developed to detect and analyze the physicochemical properties of porous particles. Electron microscopy [6,7], N2 absorption isotherms [8], and mercury instruction proximity [9] are considered the typical methods to characterize pore structure at the microscale, but suffer from long sampling time, complex operation and potentially altered particle properties. AFM [10] and NMR cytophotometry [11] have high measurement accuracy but a limited pore size measurement range. Small-angle X-ray [12] scattering can obtain abundant structural information; however, it requires complex numerical analysis methods and eliminating interference effects. Compared to these methods, polarization scattering measurement exhibits unique advantages and has been widely applied in aerosol particle detection [13].

On the one hand, it can directly measure particles over a wide scale range of pore size without any complex sample prep. On the other hand, multi-angle polarization measurements can increase the dimensionality of information, which can reflect differences in the microstructural characteristics of aerosol particles. Moreover, multi-angle polarization measurements can enable in situ, rapid, accurate and high-throughput analysis for aerosol particles, as demonstrated in previous studies by our team [14,15,16,17,18].

In addition to experimental measurement, theoretical modeling and numerical calculation is an effective approach to understand the light-scattering properties of suspended particles. Mie theory is commonly used to describe the light scattering characteristics of a single, optically isotropic sphere, but it does not apply to particles with irregular shapes or optical anisotropy. To solve this, more light-scattering simulation methods have been developed, such as Finite Difference Time Domain (FDTD) [19], Finite Element Method (FEM) [20], Multiple Sphere T-Matrix Method (MSTM) [21], and Discrete Dipole Approximation (DDA).

The DDA method can calculate the scattering and absorption properties of arbitrarily shaped objects, which can be inhomogeneous and anisotropic [22]. In addition, compared with other existing spatial discretization modeling methods, such as FDTD or FEM, DDA is not limited by far-field boundary conditions and requires less computational memory [23]. On the other hand, DDA requires solving a large system of linear equations in modeling, which demands the platform’s high computational power. Especially for objects with large size and high permittivity, even if the operation can be accelerated by Fast Fourier Transform (FFT), the speed of convergence during iteration is still slow. In addition, the electric field inside the object with high permittivity would exhibit oscillations that reduce the accuracy of DDA simulations. In addition, DDA is not applicable to metal and perfect conductors [24].

For porous particles, various models have been proposed. For example, Lumme [25] and Vilaplana [26] randomly remove a certain number of dipoles (scattering units) from the original solid model to generate porous particles with arbitrary porosity. On this basis, Sen [27] developed a novel algorithm to ensure the connectivity of remaining dipoles after random removal and researched the influence of porosity on the polarized properties and color of dust particles from the coma. Conversely, Zubko [28] constructed a debris particle model with a hierarchical structure by adding dipoles and vacuum in a certain proportion to the lattice cells inside a sphere. Lindqvist [29] used a similar method to generate a simulated vesicular volcanic ash particle model by adding Gaussian random spheres with irregular shapes to a cluster of spheres. In addition, Tang [30] proposed a porous fly ash particle model based on the particle superposition model and studied the effect of the pore-dispersed distribution on the radiative properties of porous particles.

In particle modeling research, the primary considerations are the particle shape, size, pore size, and porosity; other factors that should be taken into account include the authenticity in terms of openness, connectivity, and dispersion distribution of pore structure, especially for the case of the modeling simulation involved in the inverse problem of particle parameter inversion. In this paper, we propose a general porous particle modeling method based on an overlapping sphere model, which allows independent and flexible adjustment of the average pore size and porosity closer to the genuine morphology and structure of porous particles. The polarization scattering characteristics of porous particles are investigated by the discrete dipole approximation (DDA) method. Furthermore, we use a single-particle multi-angle polarization measurement device to obtain multi-dimensional polarization scattering indexes of certain porous particles. By combining theoretical calculation and experimental results, we exact corresponding polarimetric indexes to analyze the effect of the pore size and porosity on the polarimetric properties. Based on this data, our study achieves pore particle identification, content extraction under mixed conditions, and quantitative inversion for porosity and average pore size.

## 2. Materials and Methods

### 2.1. Materials

**Figure 1:**
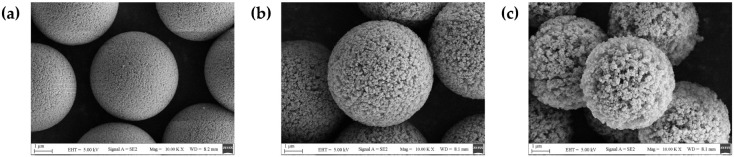
The SEM images of three polymer samples. (**a**) Unips5-100Å; (**b**) Unips5-300Å; (**c**) Unips5-1000Å.

Four types of particles with different pore sizes and porosities are used as experimental samples in this study, and their properties are shown in Table 1. PSL spheres (Da e, Tianjin, China) are solid particles without pore structure, while the other three polymer samples are porous microspheres (Unips5-100Å, Unips5-300Å, and Unips5-1000Å, NanoMirco, Suzhou, China). Their sizes are all five μm, and they have a similar coefficient of variation (C.V.) of particle size distribution, which is less than 0.028. The complex refractive index m for all particles is 1.59 + 0i. Figure 1 shows the SEM images of three polymer samples.

### 2.2. Experimental Setup

**Figure 2:**
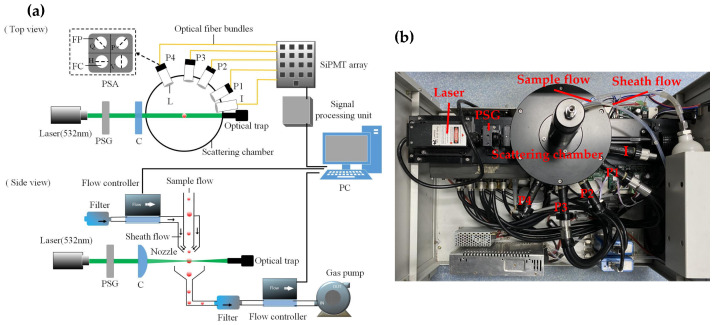
(**a**) Schematic of the experimental setup. Laser—solid-state laser system with a wavelength of 532 nm (MSL-III-532, Changchun New Industries Optoelectronics Tech, Changchun, China). PSG—polarization state generator; C—cylindrical lens; I—light intensity channel at 10°; L—lens; P1–P4—polarization state analysis channel at 30°, 60°, 85°, and 115°; PSA—polarization state analyzer; FP—film polarizer; FC—fiber core; H—horizontal polarizer film; V—vertical polarizer film; P—45° polarizer film; Q—135° polarizer film; SIPMT—silicon photomultiplier tube. (**b**) Photograph of the measuring instrument.

The schematic of the experimental setup is shown in Figure 2a. In the optical system, a horizontally polarized laser beam with a wavelength of 532 nm passes through a polarization state generator (PSG) composed of a polarization beam splitter and a quarter-wave plate, where the incident light is modulated into three polarization states: horizontal linear polarization state (H), 45° linear polarization state (P), and right circular polarization state (R). The polarized light continues to pass through a cylindrical lens to generate a light sheet with a width of 1 mm and a thickness of 0.04 mm and then is focused on the sample flow of the suspended particles. The stray light is absorbed by the scattering chamber with high-absorption materials, and the forward non-scattering light is absorbed by the light trap at the terminal of the device.

As shown in the side view of Figure 2a, the gas flow generation system consists of two flow controllers, an air pump, and a special sheath nozzle. The sheath nozzle is designed with a dual-layer structure, where the outer layer is the channel for the sheath flow, and the inner layer is the channel for the sample flow. During the experiment, particles in the sample flow are wrapped by the injected sheath flow to smoothly pass through the center of the light detection area one by one. The velocity of total gas flow is limited to 2 L/min, composed of the sample flow rate of 1 L/min and sheath flow rate of 1 L/min.

The light scattered by an individual particle and its polarization state are detected and analyzed at four angles of 30°, 60°, 85°, and 115°. Each detection channel is equidistant from the center of the scattering surface and equipped with a polarization state analyzer (PSA), which consists of four linear polarizers oriented at different angles of 0°, 45°, 90°, and 135°). Meanwhile, an optical intensity detection channel is added at 10° to detect the trigger signal and obtain the size and number of particles. Each detection channel is connected to a silicon photomultiplier tube array through optical fibers. Then, the signal is converted and amplified by a signal processing unit and transmitted to a high-speed data acquisition card. The sampling rate of the data acquisition card is up to 1 M/s, which can record the scattering pulse signals of 2000 suspended particles per second.

### 2.3. Stokes Vector and Polarization Indexes

The scattering light intensity of one individual detected particle at each polarization detection channel is $I={\left[I_{0^{\circ }},I_{45^{\circ }},I_{90^{\circ }},I_{135^{\circ }}\right]}^{T}$, and the Stokes vector can be calculated in Equation (1):

(1) $$S_{out}=\left[\begin{array}{c} s_{0}\\ s_{1}\\ s_{2} \end{array}\right]=\left[\begin{array}{c} I_{0}+I_{90^{{}^{\circ}}}\\ I_{0}-I_{90^{{}^{\circ}}}\\ I_{45^{{}^{\circ}}}-I_{135^{{}^{\circ}}} \end{array}\right]$$

Polarization indexes *Hdop* and *Pdop* can be obtained by normalizing *s*_1_ and *s*_2_ with *s*_0_, as shown in Equation (2):

(2) $$Hdop=\frac{s_{1}}{s_{0}},Pdop=\frac{s_{2}}{s_{0}}$$

In this paper, we employ six Stokes polarization indexes, including *H-Hdop*, *H-Pdop*, *P-Hdop*, *P-Pdop*, *R-Hdop*, and *R-Pdop*, where the letters before and after the symbol “-” respectively represent the polarization states of the incident light and the scattering light. There are a total of twenty-four polarization indexes for each particle by synchronous measurement at four scattering angles.

### 2.4. System Calibration

**Figure 3:**
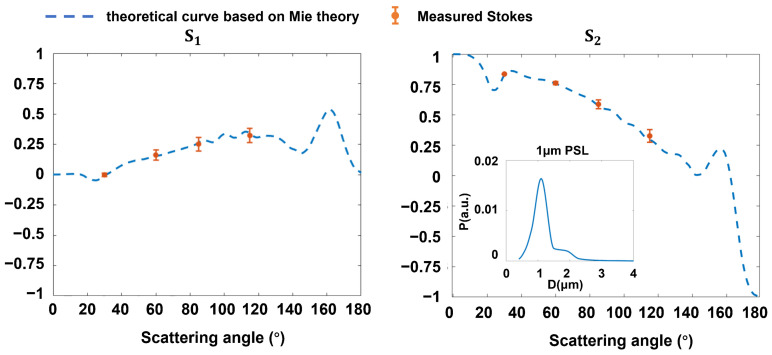
The calibration results of monodisperse PSL sphere. The subplot in S_2_ shows the curve for the optical particle size distribution of PSL.

To validate the measurement accuracy, we used 1 µm polydisperse polystyrene latex (PSL) as a standard particle sample to calibrate our experimental device. We recorded the experimental measured Stokes vectors at four scattering angles. As shown in Figure 3, the curves represent theoretical values using Mie theory, and the boxplots represent the experimental values from polarization scattering measurements.

For Stokes components *s*_1_ and *s*_2_, the average standard deviations at four detection angles are both less than 0.05. The consistency between experimental data and simulation results validates the reliability of our device. Specifically, to mitigate the influence of particle spatial position and orientation on the individual particle scattering signal, the experimental results were averaged over 200 data points. In addition, a narrow particle size distribution led to oscillation in theoretical curves. Therefore, we employed a Gaussian distribution centered at 1 µm as particle size distribution to modify the Mie scattering algorithm, which can reduce the oscillation of scattering signals.

### 2.5. Modeling of Porous Particles

In this work, we propose a general porous particle model based on an overlapping sphere model. The modeling process can be described as follows.

(1) According to actual particle size, we first generate an array of *N_cube_* elements located on a cubic lattice. *N* dipoles are randomly added to unit cells of the cubic lattice. Then, we loop through each element within the lattice and compute the Euclidean distance *D* between the element and its nearest dipole. By comparing *D* with pre-defined radius *R_ad_*, the pore is created when $D\geq R_{ad}$, while a new dipole is added when $D<R_{ad}$. Therefore, the initial porous particle model is constructed by overlapping some spheres with a radius of *R_ad_*.
(2) Regulate the number of *N* to guarantee that the porosity of the initial model *p_temp_* is equal to the target porosity *p*. The value of *p_temp_* can be calculated by Equation (3).
  (3) $$p_{temp}=\frac{N_{hole}}{N_{cube}}$$
  where *N_hole_* is the number of pores. In this way, a cubic porous particle model with a specific porosity.
(3) To calculate the average pore size *r_temp_*, we employ the watershed transformation method to extract the pore regions within the model. Then, we generate spheres with the same number of elements as the segmented pore areas, and the radius of the equivalent sphere is considered as the pore size. The average pore size *r_temp_* is the result of averaging the radii of all equivalent spheres.
(4) The cubic model is cut into the target shape. Finally, we obtain a porous particle model with specific shape, size, porosity, and average pore size *r*.

**Figure 4:**
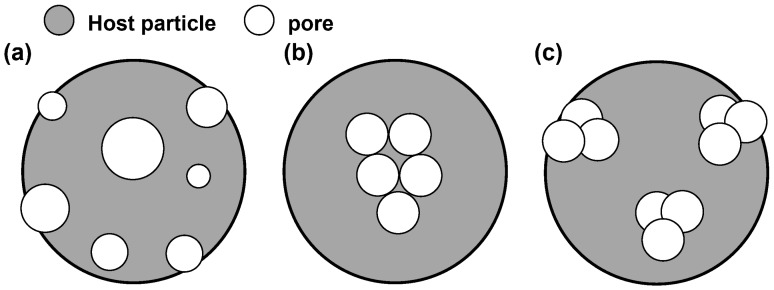
Three porous particle models: (**a**) dipole stochastic removal model. Circles with different diameters represent holes with different average pore sizes that cannot be preset; (**b**) particle superstition model. Pore structures are interconnected and located at the center of mass of host particle; (**c**) overlapping sphere model. It has a more dispersed pore distribution than some of the holes that exist at the model surface and form open pore structures.

In addition, the dipole stochastic removal model [25,27] and particle superposition model [30] are the other two common modeling approaches for porous particles. The former generates porous aggregate structures by stochastically removing some dipoles from a compact pseudo-sphere; the latter creates pores in the host particles by spherical fractal aggregates with a specific monomer radius. Although the principle and process of the dipole stochastic removal model are simple, it can only independently set the porosity rather than the pore size, as shown in Figure 4a. The particle superposition modeling method allows regulation of the number of pores and monomer radius to establish porous particle models with different porosities and pore sizes. However, when the monomer radius is considered as pore size, most pores are inescapably concentrated around the center of mass, constructing closed inner holes with heterogeneous distribution. Moreover, all pores obtained by this method are connected due to a fractal structure, which deviates from the properties of actual porous particles.

Compared with the above two methods, the proposed model is more flexible, convenient, and broadly applied. Various particles’ morphology and physical properties allow for flexible modeling parameter settings, including size, shape, pore size, and porosity, through a set of algorithms that can improve modeling efficiency. Furthermore, overlapping spheres are randomly generated at delimited locations within the model to obtain more dispersed pore structures instead of closed inner holes, which are more aligned with the properties of real porous particles. Figure 4b,c show the difference in the distribution and openness of pore structures between the particle superposition model and the overlapping sphere model.

### 2.6. Simulation Calculations

In this work, the light scattering process of porous particles was simulated by using NMDDA [31] based on discrete dipole approximation (DDA). Our research team developed the algorithm, which can achieve rapid computation with the help of GPU acceleration. To ensure the validity of the DDA method, the size of dipoles *d* should be smaller than the wavelength of incident light. It requires $\left|m\right|kd<1$, where *m* is the complex refractive index of the particle and k=2π/λ is the wavenumber. In addition, the number of dipoles *N* should be large enough to describe the particle shape satisfactorily. Consequently, we set $\left|m\right|kd$ it to 0.8 and *N* to 10^8^ during the simulation.

**Figure 5:**
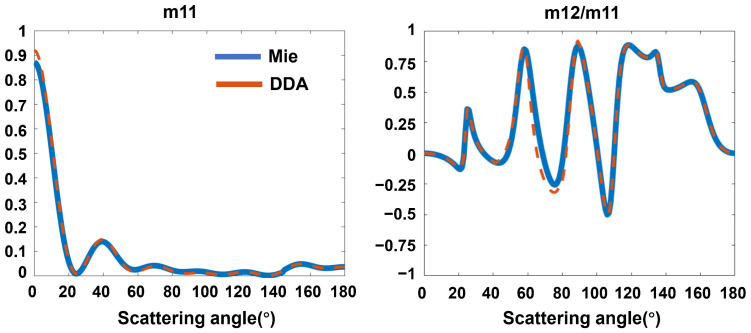
Comparison of the calculation results based on DDA and the Mie theory.

We compare the scattering characteristics of a 1 μm solid particle calculated by DDA with the calculated results based on Mie theory, as shown in Figure 5. The Mueller matrix elements *m*_11_ and *m*_12_/*m*_11_ calculated by DDA almost agree with those calculated with the Mie theory, and the error is less than 0.01, which confirms the reliable accuracy and stability of numerical calculation based on DDA.

### 2.7. Methods for Estimating the Proportion of Components of Mixed Particles

Based on the difference in polarization properties between porous and solid particles, we propose a method to estimate the proportion of each component in a mixture of them. 

The mixture of multi-class suspended particles can be described as *β* (*β_1_*, *β_2_*, …, *β_n_*)*. f_j_* is the proportion of the jth component, given by Equation (4).

(4) $$f_{j}=\frac{Num(\beta_{j})}{Num(\beta )}$$

The mean value of multi-dimensional polarization indexes of the multi-class particles *d* can be calculated by Equation (5).

(5) $$d=\sum_{j=1}^{n}\alpha_{j}f_{j}$$

where *α_j_* is the mean value of multi-dimensional polarization indexes of some certain known particle type.

As shown in Equation (6), the proportions of each type can be obtained by solving a series of linear equations by the non-negative least squares.

(6) $$\underset{f}{\min}\left\|{\left.\alpha \cdot f-d\right\|}_{2}^{2},f>0\right.$$

The sum of squared errors (SSE) is used to describe the accuracy of the analysis as Equation (7).

(7) $$SSE(\%)=100\times \sum_{j=1}^{n}{\left(f_{e,j}-f_{p,j}\right)}^{2}$$

where *f_e_*_,*j*_ is the true proportion of each source and *f_p_*_,*j*_ is the extracted proportion of each source.

## 3. Results and Discussion

### 3.1. Theoretical Simulation on Pore Size and Porosity

Porosity and pore size are two important characteristics of porous particles, and this section shows the influence of these two factors on the polarization scattering properties of porous particles. Based on the models in Section 2.5, we can separately extract the Mueller matrix *M* corresponding to different pore sizes and porosities. By Equations (2) and (8), we further calculate the Stokes polarization indexes at different scattering angles from 0° to 180°.

(8) $$S_{out}=M\cdot S_{in}$$

where *S_in_* represents the Stokes vector corresponding to horizontally, vertically, and 45° polarized incident light.

**Figure 6:**
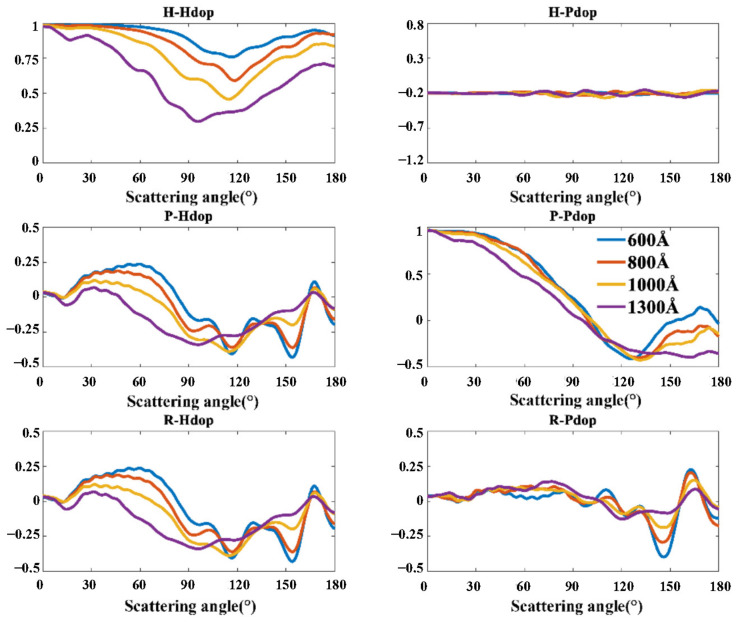
Stokes polarization indexes of porous particles with different pore sizes.

In the simulations, the particle size is set to 5 μm, and the complex refractive index is set to 1.59 + 0i. We first set the porosity to 30.3% and calculate the Stokes polarization indexes of porous particles with the pore size from 600 Å to 1300 Å, as shown in Figure 6.

The variation of pore size has a significant impact on the *H-Hdop* index. The values of *H-Hdop* at all scattering angles decrease with the increasing pore size. *P-Hdop*, *R-Pdop,* and *R-Hdop* index are also sensitive to the difference in pore sizes. The effects of pore size on the *P-Hdop* and *R-Pdop* index are mainly reflected at the forward scattering angle range, while the effect of pore size on the *R-Hdop* index is mainly reflected at the backward scattering angle range. Specifically, when the scattering angle is less than 110°, the *P-Hdop* and *R-Pdop* index decrease with increasing pore diameter. When the scattering angle is more than 150°, the *R-Pdop* index also decreases with increasing pore size.

**Figure 7:**
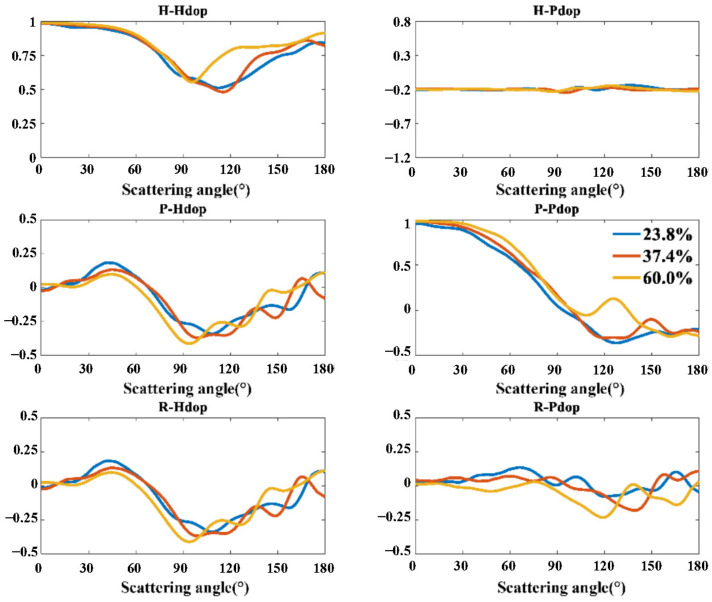
Stokes polarization indexes of porous particles with different porosities.

Next, we set the pore size to 1000 Å and calculate the Stokes polarization indexes of porous particles with porosity at 23.8%, 37.4%, and 60%, respectively. The results are shown in Figure 7.

Within the scattering angle from 30° to 75°, the *P-Pdop* index increases with the increasing porosity, while the *P-Hdop*, *R-Pdop*, and *R-Hdop* decrease.

As shown in Figure 6 and Figure 7, the correlation between polarization indexes and particle properties is not consistent at different scattering angles. Both experimental results and theoretical calculations show that at some angles, good monotonicity can be found between polarization indexes and particle characteristics, such as pore size, while at other angles, the same polarization index can be insensitive to particle characteristics or change irregularly. Optical measurements based on the single-particle scattering principle usually need to select the suitable scattering angle to obtain the optimal analytical ability of the detected particle. In past work, we usually use the standard particle test or theoretical simulations to observe the monotonic and non-monotonic angular range of the polarization scattering angle distribution and then explain the rationality of setting the measured angle. Similar analyses and results can be found in some of our previous papers [15,32].

The theoretical calculations show the feasibility of characterizing two key parameters of porous particles from multi-scattering angle polarization vector indexes. It provides a theoretical basis for subsequent particle identification and quantitative inversion for pore parameters.

### 3.2. Experimental Identification of Porous Particles

In this section, the measured polarization indexes are employed to identify PSL spheres and the other three polymer porous microspheres.

**Figure 8:**
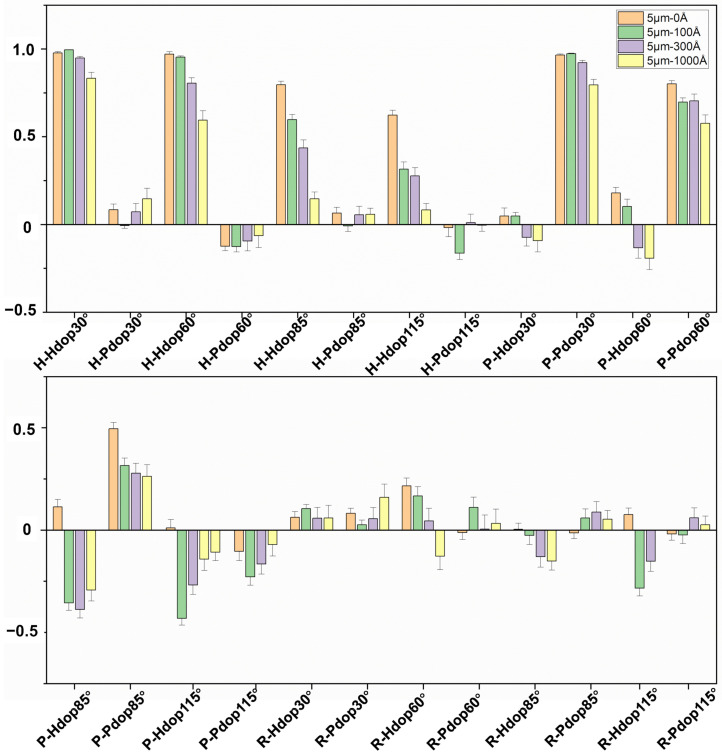
The mean values of 24 Stokes polarization indexes measured from the four samples at four detection angles (30°, 60°, 85°, and 115°).

Figure 8 shows the statistical results of the Stokes polarization indexes measured from the four samples at four detection angles (30°, 60°, 85°, and 115°). Each bar represents the average value of 200 data points. According to our previous studies and theoretical calculations, the differences in these polarization indexes reflect the differences in the microphysical characteristics of the particles, such as pore size and porosity.

**Figure 9:**
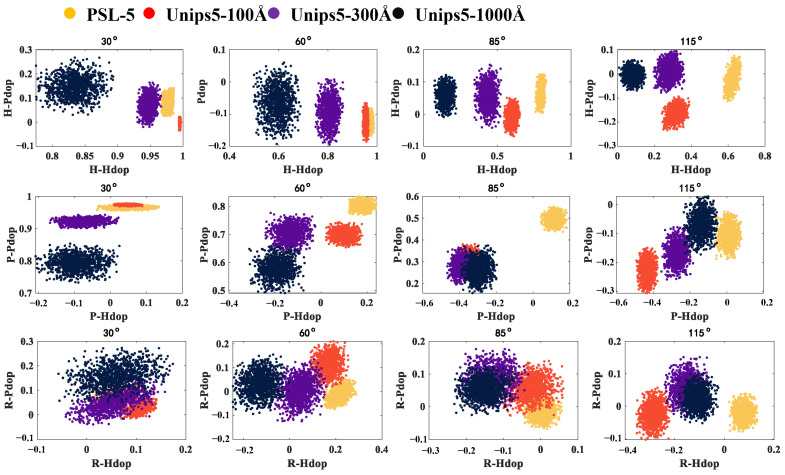
The scatter diagram of the polarization indexes for the four samples under three incidence polarization states (H, P, and R).

Moreover, the scattering plot of the Stokes polarization indexes for the four samples under three different incident polarization states (*H*, *P*, and *R*) is shown in Figure 9.

The recognition ability of stokes indexes at different scattering angles and different incidence polarization states is different. The polarization indexes under *H* incidence polarization states show stronger discrimination than those under *P* and *R* incidence polarization states. Specifically, the forward (30° and 60°) *H* polarized indexes are suitable for distinguishing among three porous particles rather than solid PSL spheres, while the backward (85° and 115°) *H* polarized indexes are better at discriminating all four samples. Moreover, the *H-Hdop* indexes at all four detection angles decrease with the increasing pore size, which is consistent with theoretical calculations.

In addition, the indexes corresponding to *P* incident polarization states can be used to distinguish particles at specific scattering angles, such as 30° and 115°. The *P-Pdop*(30°) index of porous particles decreases with increasing pore size, which is also in agreement with the simulation results.

In contrast, it can be seen that four particles are almost inseparable in polarization indexes under *R* incident polarized light. According to the simulating calculation-based DDA model, *R* indexes would decrease whether the pore size or porosity increases, and the porous particles used in the experiment are either large pore size with small porosity or small pore size with large porosity. Therefore, the influence of the two key parameters on *R* indexes would restrain each other, leading to small differences in *R* indexes for the four particles.

### 3.3. Proportional Analysis of Mixed Suspended Particles

**Figure 10:**
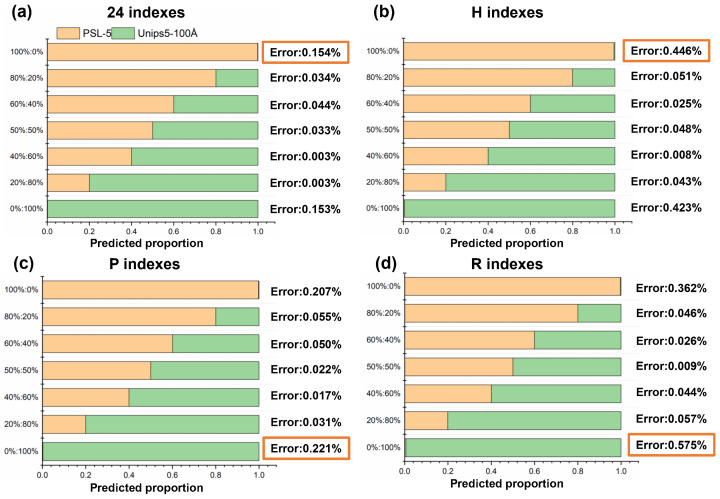
The results and errors of the proportional analysis for hybrid particles using different Stokes polarization indexes (**a**) 24-dimensional polarization indexes; (**b**) Polarization indexes under H incident polarized light; (**c**) Polarization indexes under P incident polarized light; (**d**) Polarization indexes under R incident polarized light. The error in orange frame represents the maximum error.

**Figure 11:**
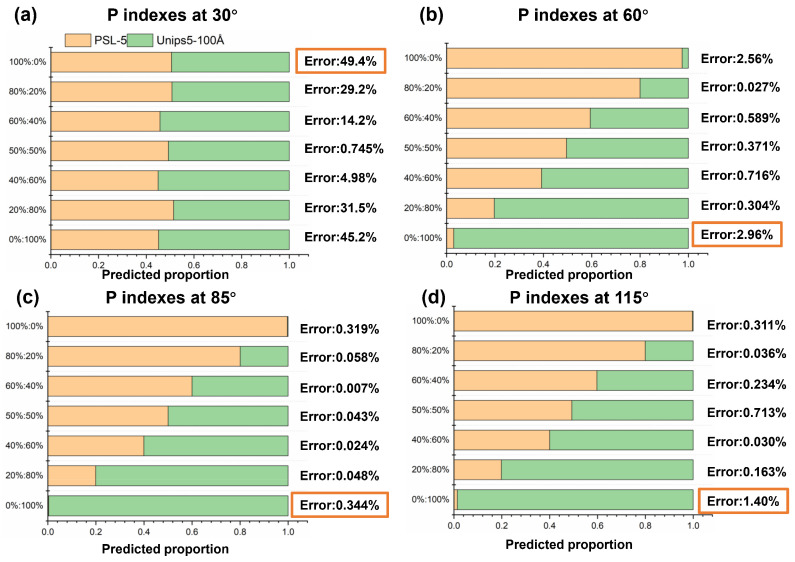
The results and errors of the proportional analysis for hybrid particles using *P* Stokes polarization indexes at four scattering angles (**a**) 30°; (**b**) 60°; (**c**) 85°; and (**d**) 115°. The error in orange frame represents the maximum error.

To further extract some specific polarization indexes to discriminate solid and porous particles, we employ the mixed particles composed of PSL-5 and Unips5-100Å. Two particles are mixed with seven proportions, such as 0:1, 0.2:0.8, 0.4:0.6, 0.5:0.5, 0.6:0.4, 0.8:0.2, and 1:0. The predicted proportions based on different polarization indexes and the comparison with the preset proportion are shown in Figure 10 and Figure 11. The error within the red box is the maximum analytical error.

As shown in Figure 10, the classification of porous particles has different accuracy using different combinations of polarization index. The highest accuracy is achieved using the 24-dimensional polarization indexes, with an error of 0.154%. Based on eight-dimensional polarization indexes using *P* linear polarized incident light, the analysis accuracy is also very high, with an error below 0.3%. Although the complete 24-dimensional polarization indexes can provide the best analytical result, experiment operation involving three types of incident polarized light is complex. To minimize the complexity of the measurements, we applied the polarization indexes using *P* linear polarized incident light to evaluate the classification ability in the following study.

Next, we compare the analytical results derived from the *P* polarization indexes at four angles, as shown in Figure 11a–d.

Compared with the above results using multi-angle polarization indexes, the accuracy decreases relatively when polarization data is measured at only one single detection angle. The error at the scattering angle of 30° is much higher than errors at the other three angles, which can be explained by results in Section 3.2. As shown in Figure 9, it is difficult to discriminate PSL-5 and Unips5-100Å by *Pdop* at small forward scattering angles (30°). The optimal detection angle with the best accuracy is 85°, and its error is 0.344%. Therefore, the two polarization indexes at 85°, including *P-Hdop*(85°) and *P-Pdop*(85°), are optimal indicators to achieve accurate proportional analysis of mixed particles.

### 3.4. Inversion of Porosity and Average Pore Size

The simulation and experimental results confirm the sensitivity of polarization scattering characteristics of porous particles to the variation of pore size and porosity, which implies the potential to deduce these two factors by polarization indexes. However, the intertwined influence of pore size and porosity poses a challenge to parameter inversion. As presented in Figure 7, the effect of porosity on *H-Hdop* is insignificant at forward scattering angles (30° and 60°), and *P-Hdop*, *P-Pdop*, *R-Hdop*, and *R-Rdop* show monotonic changes with pore size or porosity. Therefore, we use these polarization indexes to achieve quantitative inversion of pore size and porosity in two steps. The details of the inversion algorithm are as follows:

(1) According to DDA simulation, we first calculate the two Stokes indexes *H-Hdop*(30°) and *H-Hdop*(60°), corresponding to different pore sizes that vary from 0 nm to 10 nm with a step of 0.1 nm. Two indexes are represented $S_{T,HH}(\theta ,r)$, where *T* represents the theoretical value, *θ* is the scattering angle, and *r* is the pore size. Next, the predicted pore size of target particles can be derived from minimizing the norms between the theoretical value and measured data $S_{M,HH}(\theta ,r)$, as expressed in Equation (9).
  (9) $$\chi_{r}^{2}(\theta ,r)=\underset{r}{\arg\min}{\left\|S_{T,HH}(\theta ,r)-S_{M,HH}(\theta ,r)\right\|}_{2}$$
  where the optimal estimate of the pore size corresponds to the minimum value of $\chi_{r}^{2}(\theta ,r)$.
(2) Based on the pore size obtained in step 1, we further extract the theoretical values of the polarization index $S_{T}(\theta ,p)$ corresponding to different porosities *p* that change from 0 to 100% with a step of 0.1%. The polarization indexes contain *P-Hdop*(30°, 60°), *P-Pdop*(30°, 60°) and *R-Pdop*(30°, 60°). Then the least squares method is used to calculate the minimum difference between the theoretical values and measured data $S_{M}(\theta ,p)$, and obtain the predicted porosity, as shown in Equation (9).
  (10) $$\chi_{p}^{2}(\theta ,p)=\underset{p}{\arg\min}{\left\|S_{T}(\theta ,p)-S_{M}(\theta ,p)\right\|}_{2}$$

The measured data of the three porous particles (Unips5-100Å, Unips5-300Å, and Unips5-1000Å) are used to assess the feasibility of the inversion algorithm, and the results are shown in Table 2.

**Table 2:** The inversion results of average pore size and porosity.

	Actual Pore Size (nm)	Predicted Pore Size (nm)	Actual Porosity (%)	Predicted Porosity (%)
Unips5-100Å	10	9.2	62.0	55.8
Unips5-300Å	30	31.1	30.3	32.1
Unips5-1000Å	100	102.2	23.8	24.6

The deviations of inversion pore size for Unips-300Å and Unips-1000Å are less than 4%, and the errors of inversion porosity are less than 6%. Comparatively, the predictive bias for Unips-100Å is slightly higher, with a porosity of about 8% and a porosity of about 10%. The increase of inversion error for particles with small pore sizes is probably caused by the limitation of the model accuracy. When modeling, the particle model must be large enough to generate a pore structure with small pore sizes, and it significantly increases the model complexity. Therefore, after weighing up the computing power of our platform, we increase the distance between dipoles and decrease the accuracy of the model with a small pore size, which can introduce errors into the simulation.

Moreover, since the stepwise inversion method is employed to extract the porosity based on pore size, the inversion deviation of pore size can increase the error of porosity inversion. Simultaneous inversion of porosity and pore size would reduce the errors, and it is a worthy improvement for our algorithm in the future. In addition, the oscillation of polarization indexes in DDA simulation is also a source of inversion deviations.

**Figure 12:**
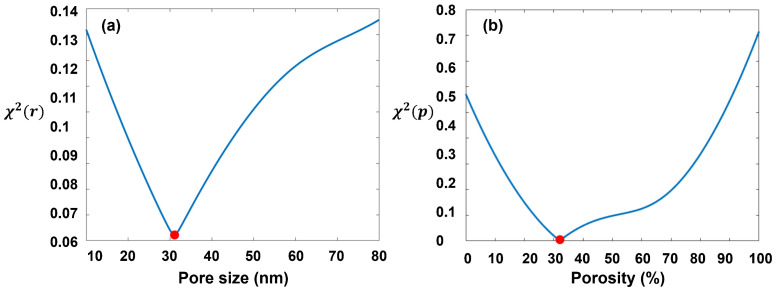
The differences between measured data and theoretical curve. (**a**) pore size; (**b**) porosity. The red dots are the minimum norms between the theoretical value and measured data at the target inversion pore size and porosity.

Figure 12 demonstrates the inversion process of Unips5-300Å.

Generally, the consistency between the inversion results and the reference data proves the feasibility and accuracy of our inversion algorithm, and it also indicates the potential of our method to characterize the micropore structure of suspended particles. 

## 4. Conclusions

In conclusion, to study the effect of pore size and porosity on the radiative properties of porous particles, we establish a spherical porous particle model based on the overlapping sphere modeling method and calculate Stokes vector indexes at different scattering angles by DDA. The simulation results show that polarization indexes change monotonically with increasing pore size or porosity at some specific scattering angles. For example, the *H-Hdop* index decreases with increasing pore size at all angles, while the same variation of *the P-Hdop* index and *R-Hdop* index can only be seen at angles less than 110°or more than 150°. For porosity, within the scattering angle from 30° to 75°, the *P-Pdop* index increases with the increasing porosity, while the *P-Hdop*, *R-Pdop*, and *R-Hdop* decrease.

Furthermore, the experiments of three porous polymer particles and a PSL sphere confirmed the validity of the identification and characterization of porous particles by a multi-angle polarization measurement system. Specifically, the backward *H* indexes (85° and 115°) and *P* index at 30° or 115° can distinguish four samples, while the *P* index at 85°is more suitable to distinguish solid spheres from porous particles. Based on these, an accurate component content analysis of multi-type mixed porous particles can be achieved using a polarization index system. Specifically, the maximum analytic error using a complete 24-dimensional polarization indexes is 0.154%, and the maximum error using partial indexes (*P* indexes at 85°) is 0.344%.

Finally, based on DDA simulations, we further propose a quantitative method to invert the pore size and porosity parameters of porous particles. The accuracy of the inversion algorithm can be evaluated by experiments of three polymer porous spheres. The studies show that the inversion error of particles with large pore size is less than 4% for pore size and less than 6% for porosity, which validates the feasibility and effectiveness of the inversion algorithm.

For the characterization and inversion of the pore structure of micro-scale particles, current studies still focus on Scanning Electron Microscopy (SEM) [33], Low-Temperature Gas Absorption (LTGA) [34,35] and small-angle neutron scattering [36]. Comparatively, synchronous polarization measurements using multiple angles and various polarization analyzing channels have the advantages of being in situ, being high-throughput, and having abundant information. Our research team proposed this technology, and based on this, this study conducted the identification and inversion of the microscopic characteristics of porous particles.

For now, our study’s inversion errors of porosity and pore size are slightly larger than those using traditional methods. In the next research, by applying it to more types of particle samples, expanding the DDA modeling and simulation calculation database, increasing polarization synchronization detection channels, and employing deep learning algorithms to improve the data mining of single-particle high-throughput online polarization measurements, the analysis accuracy of porous microstructures is expected to improve.

On the other hand, for theoretical calculations of irradiation properties of porous particles, current models based on FDTD [37], FEM [38], and DDA [30,39,40] have various limitations in disordered arrangement, connectivity, openness and setup independence of pore structures. In contrast, the proposed overlapping sphere model has advantages in solving these problems. Although experimental results of limited varieties of samples are presented in the paper, the proposed methods of modeling, measurement and analysis are generalized methods that can be applied to various porous particles from natural sources or artificial production, such as mineral dust, volcanic ash, combustion products generated from coal, and other porous polymer particles. In future work, we would conduct field experiments to measure more polarization data of porous aerosols from real atmospheric and emission sources and establish the database as the basis for the identification of atmospheric composition identification and source analysis.

In addition to atmospheric aerosol detection, the in situ and high-throughput polarization measurement method in this study can be used to quickly and accurately identify and character the pore microstructure properties of various porous particles, which can contribute to the development of new or improved porous materials for different applications, such as industrial catalysis, gas storage, sensing and actuation, biomedical imaging and drug delivery. Moreover, the proposed DDA modeling method for porous particles in this study can provide an effective reference for the theoretical calculation of the polarization radiative properties of single porous particles with various shapes and pore distributions.

## Figures and Tables

**Table 1:** Properties of four types of particles.

	PSL-5	Unips5-100Å	Unips5-300Å	Unips5-1000Å
Particle size	5 µm	5 µm	5 µm	5 µm
Pore size	0	10 nm	30 nm	100 nm
Porosity	0	62.0%	30.3%	23.8%

## Data Availability

Data are contained within the article.
